# Hyperbaric Oxygen Therapy for Complications in Nipple-Sparing Mastectomy with Breast Reconstruction: A Systematic Review

**DOI:** 10.3390/jcm13123535

**Published:** 2024-06-17

**Authors:** Omer A. Idris, Yaqub O. Ahmedfiqi, Abdulaziz Shebrain, Talal Al-Assil, Sabrina C. Pacione, Delour Haj, Abdelrahman D. Motan, Faroog Momani, Hanin Bzizi, Bahar Saadaie Jahromi, Ramona Meraz Lewis, Kyle Ver Steeg

**Affiliations:** 1 Department of Biological Sciences, Western Michigan University, Kalamazoo, MI 49008, USA; abdulaziz.shebrain@wmich.edu (A.S.); sabrina.c.pacione@wmich.edu (S.C.P.); abdelrahman.d.motan@wmich.edu (A.D.M.); faroog.o.momani@wmich.edu (F.M.); hanin.f.bzizi@wmich.edu (H.B.); bahar.saadaiejahromi@wmich.edu (B.S.J.); 2 Western Michigan University Homer Stryker MD School of Medicine, Kalamazoo, MI 49007, USA; yaqub.ahmedfiqi@wmed.edu (Y.O.A.); talal.alassil@wmed.edu (T.A.-A.); delour.haj@wmed.edu (D.H.); 3 Department of Educational Leadership, Research and Technology, Western Michigan University, Kalamazoo, MI 49008, USA; ramona.lewis@wmich.edu; 4 Bronson Methodist Hospital Plastic Surgery Specialists, Portage, MI 49024, USA; versteek@bronsonhg.org

**Keywords:** nipple-sparing mastectomy, hyperbaric oxygen therapy, breast cancer, complications, necrosis, ischemia

## Abstract

**Background:** Research advancing effective treatments for breast cancer is crucial for eradicating the disease, reducing recurrence, and improving survival rates. Nipple-sparing mastectomy (NSM), a common method for treating breast cancer, often leads to complications requiring re-operation. Despite advancements, the use of hyperbaric oxygen therapy (HBOT) for treating these complications remains underexplored. Therefore, we analyze the efficacy of HBOT in the post-operative care of patients undergoing NSM. **Methods:** A systematic search was conducted using PubMed, Scopus, and the Cochrane Library. Studies were assessed for eligibility using the PICO (Population, Intervention, Comparison, Outcome) framework and classified based on American Society of Plastic Surgeons (ASPS) levels of evidence. Seven studies, totaling a pool of 63 female patients, met the inclusion criteria. Among these studies, four were categorized as Level III (57.1%), one as Level IV (14.3%), and two as Level V (28.6%). These studies focused on HBOT’s role in wound healing, the successful salvage of breast reconstruction, and the optimal timing for HBOT. **Results:** This review revealed that HBOT indeed has potential for improving tissue oxygenation, vascularization, and, consequently, wound healing. It is noted that HBOT is efficacious for mitigating post-NMS complications, including infections, re-operation, flap loss, seroma, and hematoma. **Conclusions:** Overall, HBOT could be beneficial in standard post-surgical care protocols for patients undergoing NSM due to its role in mitigating common adverse effects that occur after mastectomy. Despite promising outcomes, the recent literature lacks rigorous clinical trials and well-defined control groups, underscoring the need for further research to establish standardized HBOT protocols.

## 1. Introduction

Breast cancer, a prevalent and devastating condition affecting millions of women worldwide, often requires mastectomy. This surgical approach plays a pivotal role in eradicating the disease and reducing recurrence, thereby increasing patient survival rates. Mastectomy is typically reserved for advanced stages of cancer, but is also considered in cases involving multifocal or multicentric tumors, small breast size relative to the tumor, and increasingly in gene carriers or patients who are at high risk of breast cancer. While patient preference is a factor, the course of action is ultimately determined by evidence-based medical practice [1]. Mastectomy involves the excision of cancerous breast tissue, including the nipple, areola, and sometimes nearby lymph nodes [2]. Mastectomy can be classified into three main types: total or simple mastectomy, which involves removal of the entire breast; modified radical mastectomy, which includes the excision of the breast and adjacent lymph nodes while still preserving the pectoral muscles; and skin-sparing mastectomy (SSM), which retains the skin of the breast to facilitate reconstruction [3]. Nipple-sparing mastectomy (NSM) is similar to SSM with the additional preservation of the areolar complex, representing an advancement toward more aesthetically pleasing outcomes for the patients [4]. Although considered a more patient-satisfactory procedure, NSM procedures include rigid exclusion criteria such as large breast size and breast ptosis [5]. For those who do not meet NSM criteria, alternative approaches exist. One such alternative is the wide-based inframammary fold flap with nipple preservation [5]. With the appropriate surgical technique and proper tissue profusion, the inframammary fold flap procedure shows promise, although caution is needed to prevent nipple necrosis. Given the variations in breast anatomy, patients have several options to consider when discussing surgical routes. The increasing rate of complications with SSM techniques can be attributed to the demanding nature of the procedure and the shift away from SSM unless necessary [6,7]. SSM and its variations, including NSM, are particularly challenging due to the need to preserve as much skin as possible [4]. SSM encompasses various subtypes, including NSM with either a pedicled nipple–areolar complex (NAC) or a free nipple graft, areola-saving mastectomy, and skin-reducing mastectomy with either a pedicled NAC or a free nipple graft, used when the skin envelope is excessively large. Another subtype is SSM, which removes the NAC but preserves the inframammary fold and a substantial portion of the skin envelope [8].

Another parameter investigated for performing an NSM procedure is the tumor-to-nipple distance (TND). There is no strict range for TND, as it varies depending on the guidelines consulted [9]. A retrospective study compared patients who had a TND greater than 1 cm to those with a TND greater than 2 cm and found that there was no significant difference in the rate of NAC preservation among each group. Although there is no strictly established protocol for TND, a recommended range is 1–2 cm [10]. Another retrospective study revealed similar results, concluding that TND, along with factors such as tumor size and multicentric cancers, should not be absolute exclusion criteria for NSM. However, favorable limits for these factors can be easily determined using further imaging studies, such as an MRI [9].

The primary risk factors for SSM in general, and NSM specifically, encompass hypertrophic breasts, ptotic breasts, thin skin and lack of subcutaneous tissue, excessively large areolas, and smoking [4,11].

Post-operatively, patients may experience pain, swelling, and limited arm mobility, with varying recovery times [12]. Breast reconstruction can be performed immediately or delayed until recovery, utilizing implants or body tissue. Despite the potential for recurrence, adjunct therapies, such as radiation, chemotherapy, and hormone therapy, may further reduce this risk [13]. Patients must make informed decisions by discussing mastectomy types, outcomes, and possible risks with their healthcare providers, as individual circumstances dictate the optimal surgical approach [14].

The advent of hyperbaric oxygen treatment (HBOT) has proven it to be a promising adjunct for wound healing and tissue recovery, aiming to enhance outcomes by leveraging increased oxygen levels to facilitate natural healing. This therapy relies on exposure to oxygen, which dissolves in the bloodstream, enhancing the healing process, potentially reducing the risk of infection, and promoting tissue regeneration [15]. HBOT is recognized for its ability to stimulate tissue regeneration and promote restoration. While its effectiveness in preventing extensive and primarily full-thickness tissue necrosis, particularly in ischemic tissue, both of the skin and subcutaneous, is not yet fully established, HBOT shows promise in minimizing wound healing disturbances such as dehiscence and superficial necrosis [16].

Despite the continuous advancements in breast cancer surgery and treatment modalities, the role of HBOT remains underexplored [17]. Comprehensive studies on the efficacy of HBOT in postoperative recovery and complication rates following NSM are lacking in the current literature. To bridge this gap, the goal of this systematic review is to assess the benefits and efficacy of HBOT in improving patient outcomes following NSM.

## 2. Materials and Methods

This review was conducted in adherence to the Preferred Reporting Items for Systematic Reviews and Meta-Analyses (PRISMA) guidelines and was registered with PROSPERO (ID: CRD42024498619) [18].

### 2.1. Search Strategy

A systematic search was conducted across PubMed, Scopus, and the Cochrane Library for literature published between 1965 and 2024. The search strategy was tailored to each database to effectively capture relevant studies. In PubMed, the following search query was utilized to target specific aspects of the subject matter:

(“surgery” [Subheading]) AND “Hyperbaric Oxygenation” [MeSH].

To encompass a broad range of terms related to the intervention, a search in Scopus was conducted using the following query:

((breast OR mastectomy) AND (“hyperbaric oxygenation” OR “hyperbaric oxygen treatment” OR “hyperbaric oxygen therapy”))

Finally, for the Cochrane Library, the search was streamlined to “HBOT for Mastectomy,” focusing on the most direct terms associated with the topic.

### 2.2. Study Selection

Study selections were accomplished by two independent reviewers using Covidence, www.covidence.org (Veritas Health Innovation, Melbourne, Australia) [19], a screening-management software. Conflict was resolved by involving a third reviewer. The study selection followed a format aligned with the PICO framework, as described below.

### 2.3. Inclusion Criteria

#### 2.3.1. Population

This review targeted studies involving individuals who had undergone NSM procedures.

#### 2.3.2. Intervention

Studies investigating the application of HBOT as a therapeutic intervention after NSM were included, encompassing studies exploring various HBOT regimens, dosages, and delivery methods.

#### 2.3.3. Comparison

Eligible studies included those that compared the effects of HBOT with standard care practices, alternative interventions, or the same patients before and after HBOT.

#### 2.3.4. Outcome Measures

A wide range of post-surgical outcomes were considered, including necrosis, ischemia, wound healing, infection rates, re-operation necessity, post-operative bleeding, flap loss, seroma, hematoma, delayed healing, breast skin swelling (edema), oversensitivity of the breast, pain, and fibrosis.

#### 2.3.5. Study Designs

This review included various study designs, including randomized controlled trials, non-randomized controlled trials, cohort studies, case–control studies, and observational studies.

#### 2.3.6. Publication Date and Language

Studies published in English from 1965 to 2024 were included to ensure a comprehensive understanding and feasible analysis of the findings.

### 2.4. Exclusion Criteria

#### 2.4.1. Study Design

This review excluded articles categorized as literature reviews, systematic reviews, and meta-analyses. This exclusion ensured that the conclusions drawn from the review were based on direct evidence rather than secondary analyses.

#### 2.4.2. Non-English Publications

Exclusion of studies published in languages other than English was due to possible barriers to accurately interpreting and analyzing the findings.

#### 2.4.3. Unrelated Interventions

Studies that did not specifically investigate HBOT as a therapeutic intervention after NSM were excluded. This decision was made to focus the review on assessing the efficacy and safety of HBOT within this context.

#### 2.4.4. Irrelevant Outcomes

Studies that did not assess the specified post-surgical outcomes relevant to NSM recovery and the potential benefits of HBOT were excluded.

#### 2.4.5. Duplicated Articles

Multiple copies or duplicates of the same article were removed.

### 2.5. Quality Assessment and Evidence Level Classification

To evaluate each included study’s quality, the Risk of Bias in Non-Randomized Studies of Interventions (ROBINS-I) tool (Version 1) was utilized, evaluating seven forms of bias, including confounding, selection of participants, classification of interventions, deviations from intended interventions, missing data, measurement of outcomes, and selection of the reported result [20]. Each of the seven included studies underwent categorization according to the American Society of Plastic Surgeons’ (ASPS) Evidence Rating Scale for Therapeutic Studies [21]. This scale delineates levels of evidence from I to V, descending from the highest to the lowest.

### 2.6. Data Extraction

Adhering to the PRISMA guidelines, independent authors conducted data extraction [22]. An Excel spreadsheet was developed to systematically record the key information from each study, including the title of the paper, lead author, year of publication, study design, sample size, body mass index (BMI), comorbidities, number of HBOT sessions, and time between surgery and initiation of HBOT. Surgical complications were also added to the Excel spreadsheet and divided into control and experimental groups across several parameters: necrosis, ischemia, wound healing, infection, re-operation, post-operative bleeding, flap loss, seroma, hematoma, delayed healing, breast skin swelling (edema), oversensitive breast, pain, and fibrosis.

## 3. Results

### 3.1. Summary of Selected Studies

**Figure 1:**
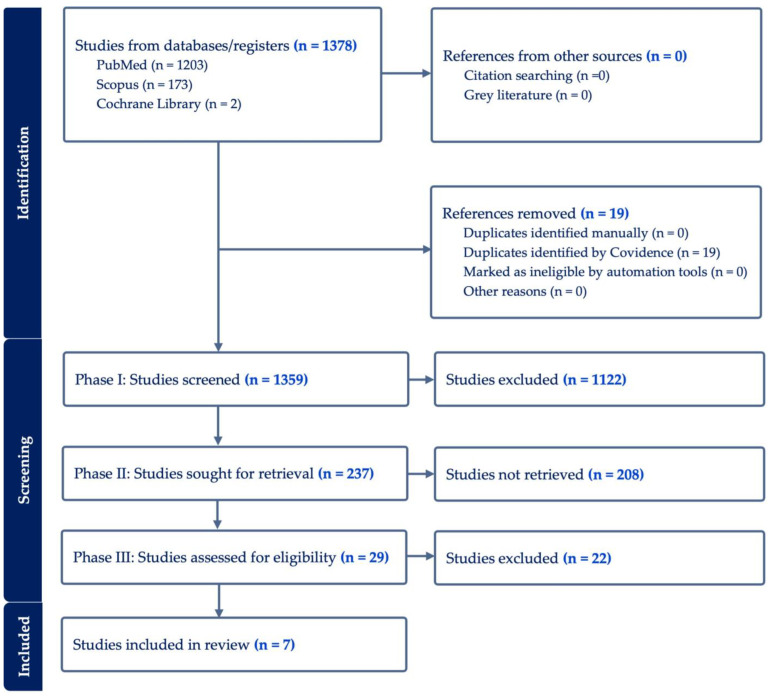
PRISMA flow diagram of study selection process.

**Figure 2:**
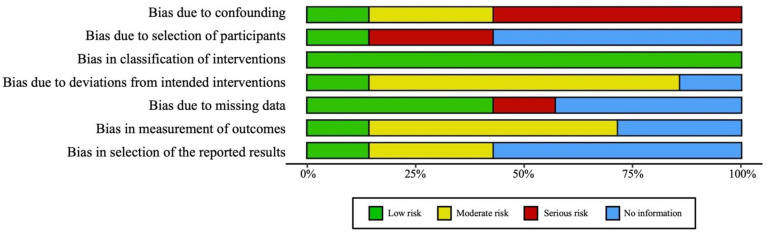
Assessment of quality and bias in the seven included studies.

**Figure 3:**
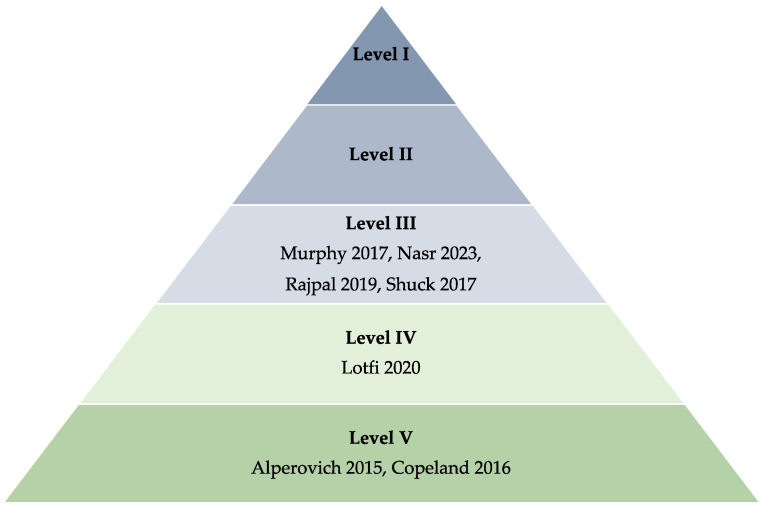
Categorization of the seven included studies according to the levels of evidence defined by the American Society of Plastic Surgeons [21,24,25,26,27,28,29,30].

A total of 1378 studies were identified for screening from PubMed, Scopus, and Cochrane library. In phase I, 1359 studies were screened through title and abstract reviews, with 237 proceeding to full-text review (phase II). In phase III, another 208 studies were excluded because they did not focus on the efficacy of HBOT in NSM. Phases I, II, and III are outlined in Figure 1. A final set of seven studies was identified for data extraction, and these were subsequently assessed for bias risk utilizing the ROBINS-I tool [20]. This comprehensive evaluation spanned eight distinct domains, as depicted in Figure 2. An unweighted summary plot of the ROBINS-I quality assessment results was generated using the Risk-of-bias Visualization (robvis) application, which enables the creation of risk-of-bias assessment figures [23]. The ASPS Evidence Rating Scale for Therapeutic Studies was used to categorize the seven included studies based on their levels of evidence [21]. As shown in Figure 3, four of the studies were classified as Level III (57.1%), one as Level IV (14.3%), and two as Level V (28.6%).

### 3.2. Data Synthesis and Analysis

The seven included studies consisted of two case reports (28.5%), one observational case series (14%), two cohort studies (28.5%), and two retrospective studies (28.5%). Across these studies, a total of 63 female patients were analyzed, all of whom had undergone an NSM procedure followed by some form of breast reconstruction surgery, had received HBOT as an intervention for surgical complications, and had previously been diagnosed with breast cancer, ensuring homogeneous data. Comprehensive details from the seven included studies focusing on HBOT with NSM are outlined in Table 1. Patients were categorized into experimental and control groups. In some studies, the experimental group consisted of patients who received HBOT, whereas the control group included patients receiving different interventions for the same complications. In other studies, the experimental condition was designated as post-HBOT, and the control condition was referred to as pre-HBOT. Pre-HBOT denotes the patient’s status post-NSM but prior to starting HBOT treatment, while post-HBOT refers to the condition of patients following initiation of HBOT.

### 3.3. Assessment of Comorbidities of the Included Patient Population

Common comorbidities were reported in 56 out of all patients analyzed across six of the seven included studies. Smoking was the most common comorbidity (6.3% of patients), followed by hyperlipidemia, hypertension, seizure, and hypothyroidism.

### 3.4. Influence of the Timing of HBOT Intervention on Managing Threatened Skin Flap Necrosis Post-NSM

**Figure 4:**
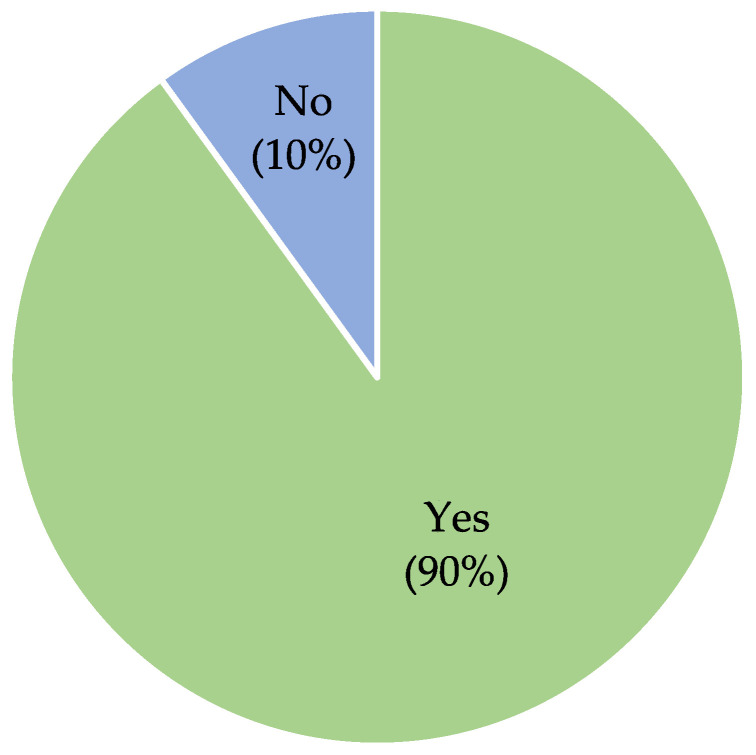
Pie chart depicting successful healing of mastectomy flaps in patients undergoing nipple-sparing mastectomy and receiving hyperbaric oxygen therapy within two days post-surgery.

The initiation of HBOT varied among the 63 patients, with specific post-operative HBOT timeframes reported for 27 individuals. Notably, 10 patients received HBOT within an optimal 48 h window following NSM. Within this early-intervention subgroup, a 90% success rate in resolving threatened skin flap necrosis (TSFN) was observed (Figure 4), with only one patient experiencing unresolved complications. This underscores the potential of HBOT to enhance surgical recovery when applied promptly. When comparing this to patients who received HBOT seven days or more post-operatively, there was a TSFN salvage rate of 88% [27].

### 3.5. Qualitative Analysis of HBOT Efficacy in Managing Flap Ischemia and Threatened Skin Flap Necrosis Post-NSM

HBOT proved to be an effective means of promoting tissue healing in a case study with a 31-year-old female patient who underwent bilateral preventive NSM and later developed TSFN [24]. The mastectomy flaps healed after 30 HBOT sessions, demonstrating the potential to save reconstructions after NSM. In another study, seven patients received HBOT following NSM and underwent immediate breast reconstruction [25]; the results indicated subjective improvements in ischemic areas and no progression to implant loss or mastectomy skin flap necrosis, suggesting that HBOT was effective in preventing complications for these patients.

In a different study [26], four patients with recurrent breast cancer who underwent NSM received HBOT. Two of them underwent both pre- and post-operative HBOT, one received only pre-operative HBOT, and one only received post-operative HBOT. Following surgery, only one (25%) of the four patients experienced superficial TSFN, which was effectively managed with topical nitroglycerin paste. This suggests that HBOT, whether administered pre- or post-operatively, may be useful in treating problems related to mastectomy.

In retrospective research [27], partial flap or NAC necrosis, along with early signs of ischemia or venous congestion, were treated with HBOT. The 88% flap salvage rate observed in this trial suggests that HBOT is useful in promoting tissue repair and managing the consequences of ischemia. 

The effectiveness of HBOT in treating TSFN and ischemia after breast reconstruction was highlighted by Rajpal et al. [28]. HBOT promoted effective healing of damaged mastectomy skin flaps, increased neovascularization, and improved flap survival. Indocyanine green angiography monitoring in conjunction with HBOT provides a useful strategy for controlling TSFN and ischemia of restored tissue.

The outcomes of TSFN and ischemia in 13 patients treated with HBOT following NSM were the subject of Shuck et al.’s study [29]. They found that using HBOT improved flap survival and healing, indicating that it is useful in preventing ischemia-related problems and accelerating tissue recovery. 

The effectiveness of HBOT in preserving tissue following bilateral NSM was examined by Copeland-Halperin et al. [30]. They demonstrated that HBOT sessions significantly improved the appearance of ischemic skin flaps. This case study highlights the advantages of early post-operative HBOT in preventing the progression of ischemia into skin flap necrosis and minimizing the need for further surgery.

### 3.6. Assessment of HBOT Efficacy for Various Surgical Complications following NSM

Re-operation: Twenty-three patients across four studies required re-operation [24,25,27,30]. Re-operation rates were higher in the pre-HBOT group than in the post-HBOT group.

Flap loss: Four patients across two studies experienced flap loss [27,28]. Flap loss rates were higher in the pre-HBOT group than in the post-HBOT group.

Sinus pain: No reported sinus pain was noted in the pre-HBOT group. One of the seventeen patients (5.9%) in the post-HBOT group experienced sinus pain [27].

While the assessments above indicate that HBOT is beneficial for various post-NSM complications, it is worth noting that the success of HBOT in experimental groups compared to the control group might represent an association rather than causation.

## 4. Discussion

### 4.1. HBOT and Its Role as Post-Operative Treatment

HBOT utilizes a hyperbaric chamber, wherein an individual breathes pure oxygen under increased pressure. It is used to address various surgical complications, notably those stemming from inadequate blood flow or oxygen delivery to tissues. This is particularly relevant for patients who undergo mastectomy and breast reconstruction who often experience complications, such as ischemia, TSFN, and loss of skin flaps [31].

A study involving 378 patients who underwent mastectomy revealed that 49% experienced post-operative complications, with 37% suffering from wound complications. Furthermore, breast reconstruction, often performed concurrently with mastectomy, significantly increases the risk of complications compared with mastectomy alone [32].

In a larger analysis of 14,894 patients, those undergoing mastectomy with immediate autologous or implant-based reconstruction showed varying rates of wound complications and infections within the first 2 years post-surgery; in addition, 2.3% of patients without reconstruction experienced wound complications compared to 4.4% with implants and 9.5% with autologous reconstruction. The rates of infection were as follows: 12.7% without reconstruction, 20.5% with implants, and 20.7% with autologous reconstruction [33]. These findings highlight the substantial impact of surgical complications in patients undergoing mastectomies and underscore the need for effective interventions to mitigate these challenges. Following established protocols for patient selection is vital when considering individuals for NSM candidacy. These protocols commonly involve criteria such as the absence of nipple involvement, tumor size, clinical examination results, lymph node involvement, and anatomical considerations [9].

HBOT has emerged as a promising solution for post-surgical complications. Retrospective studies of patients receiving perioperative HBOT in addition to mastectomy and breast reconstruction with radiotherapy have shown that HBOT is associated with a reduced risk of post-operative complications, making it a valuable adjunct in post-mastectomy care [34].

### 4.2. Mechanism of Action of HBOT and Factors Contributing to Its Efficacy

Administering HBOT at different pressures and durations raises the blood’s concentration of dissolved oxygen, which helps salvage damaged tissues, fostering a more effective healing process and thereby achieving aesthetically favorable outcomes [35]. In a study involving 48 healthy non-smoking volunteers exposed to 1 h of HBOT at mild (1.4 atmospheres absolute [ATA]) and high (2.5 ATA) pressures, elevated plasma reactive oxygen species (ROS) and catalase (CAT) levels were observed post-therapy. This demonstrated the effectiveness of HBOT in mitigating oxidative stress and bolstering antioxidant defenses [36]. This mechanism plays a vital role in promoting wound healing and preventing bacterial colonization, rendering HBOT beneficial for post-mastectomy recovery [37]. Moreover, the effects of HBOT extend to the enhancement of angiogenesis, a process vital for wound recovery and tissue regeneration. Increased production of vascular endothelial growth factor triggered by HBOT enhances blood supply to tissues [38].

In NSM, HBOT significantly reduces the incidence of ischemia and the progression to necrosis in the breast tissue, a common post-operative complication. This effectiveness was demonstrated in a retrospective analysis of 50 breasts after mastectomy, in which HBOT led to a noticeable decrease in the surface area affected by ischemia. Remarkably, 58% of cases analyzed did not require further surgical intervention after receiving HBOT, highlighting its potential to limit the need for additional surgeries by effectively managing TSFN post-operatively [39].

Moreover, the literature suggests that the timing of HBOT initiation following surgery or after the onset of ischemia, TSFN, and flap loss significantly influences treatment efficacy. Initiating therapy within 7 days post-operatively (ideally within the first 2 days) is thought to yield optimal results [24]. In our cohort, the timing of HBOT initiation ranged from half a day to 42 days post-operatively. Notably, the success rate of HBOT in resolving TSFN among patients who received HBOT within the ideal 2-day window was only 2% higher than that among those treated more than 7 days post-operatively. This comparison underscores that while timely HBOT is recommended, delaying it does not significantly compromise clinical outcomes. Such delays may be considered if they align with the patient’s preferences for additional recovery time, comorbidities, other medical priorities, social factors, or instances of loss to follow-up. This highlights that the benefits persist even with delayed treatment initiation, allowing for a degree of flexibility in clinical applications. While early treatment is preferable, patients can still benefit from HBOT if they miss the ideal 2-day post-operative period.

The exploration of HBOT across various pressures and treatment durations has shown promise in enhancing post-surgical recovery. In a study analyzing skin scores before and after HBOT, skin scores demonstrated notable improvement more often than deterioration, and over half of the patients did not need further surgery [27]. These insights support the broader adoption of HBOT in clinical settings and highlight its versatility and effectiveness across various stages of patient care.

### 4.3. Impact of Comorbidities on HBOT Efficacy in Patients Undergoing NSM

Comorbidities shared among patients often result in similar outcomes following surgical procedures, as these pre-existing health conditions significantly affect the body’s healing capabilities and responses to surgical interventions. Comorbid conditions such as prior radiation exposure, smoking, diabetes, and elevated BMI, which are recognized risk factors affecting surgical outcomes, also play a crucial role in the efficacy of HBOT for patients undergoing NSM [36]. For instance, patients with well-managed diabetes and in- range glucose levels benefit from HBOT because it enhances wound healing by boosting oxygen delivery to compromised tissues, a common issue in diabetes. Conversely, in a retrospective analysis of patients undergoing HBOT, 16 out of 107 patients with a smoking history experienced diminished benefits from HBOT. This is attributable to the adverse effects of smoking on the wound healing process. When comparing these two comorbidities, diabetes may enhance the benefits of increased oxygen delivery, whereas smoking may hinder wound healing, thus negatively affecting HBOT efficacy [40,41]. Therefore, although HBOT is a valuable adjunct therapy for managing certain complex health conditions, particularly in patients with diabetes, individualized patient assessments are necessary to predict the efficacy of HBOT in post-mastectomy recovery.

### 4.4. Role of HBOT in Surgical Complications following NSM

Complications following mastectomy, including delayed wound healing, infection, ischemia, TSFN, seroma, hematoma, radiation-induced damage, and lymphedema, pose significant challenges, with HBOT emerging as a promising therapy for mitigating these issues. The effectiveness of HBOT for managing post-NSM complications has been demonstrated, particularly in terms of improving clinical outcomes and diminishing the need for further surgery [42].

NSM procedures are associated with specific complications, notably ischemia, TSFN, and/or NAC necrosis, with prevalence rates ranging from 2–52% [27,29]. Preservation of the NAC, with a significant aesthetic advantage, also presents the highest risk for complications such as TSFN, which can lead to implant exposure and adversely affect the final aesthetic outcome [25]. Complications associated with NSM arise primarily due to impaired perfusion of the mastectomy flap, highlighting the necessity of powerful and efficient oxygenation therapies like HBOT. By enhancing oxygen delivery to tissues, HBOT fosters an environment conducive to healing, stimulates angiogenesis, reduces inflammation, promotes the repair of damaged cells, and reduces the likelihood of TSFN [31]. Therefore, earlier initiation of HBOT (at the onset of ischemia) has been linked to higher success in the management of TSFN, close to 90%, with a decrease in the need for other surgeries or interventions [27].

The reduction in surgical complications, such as infections, re-operation, flap loss, seroma, and hematoma, post-HBOT reflects its broad-spectrum efficacy in managing NSM complications. This aligns with the observed decrease in the compromised skin areas and underscores HBOT’s role in improving clinical outcomes and reducing the need for additional surgeries. Despite its minimal side effects, HBOT is safe and efficient for treating both acute and chronic wounds [43].

### 4.5. Safety of HBOT

Several studies involving patients treated with HBOT for tissue ischemia after breast reconstruction surgery have reported no adverse effects or toxicities, indicating a generally favorable safety profile [28,34,44]. However, the literature also documents instances where HBOT was discontinued due to adverse effects, as observed in the case of a 71-year-old woman experiencing wheezing and oropharyngeal mucosal dryness and another patient who developed a hyperoxic seizure following 20 HBOT sessions [30,44]. These incidents highlight the need for individualized treatment plans and careful patient selection and monitoring during HBOT.

Additionally, the most reported side effect in a study involving 44 patients was difficulty equalizing ear pressure [27]. In some instances, this leads to severe sinus pain and discontinuation of HBOT [44]. Other reported side effects include myopia, fatigue, and temporary vision changes, with only a small proportion of patients experiencing long-lasting vision changes post-HBOT [44]. However, some of these conditions may be mitigated using nasal decongestant sprays or myringotomy tubes. Nevertheless, absolute contraindications for HBOT, such as the use of doxorubicin or mafenide acetate and untreated pneumothorax, must rigorously adhere to [45]. Finally, for those who experience discomfort in enclosed spaces (claustrophobia), alternatives such as psychological support or the use of larger multipatient chambers are available [46].

Despite documented side effects such as sinus pressure and ear pain, their frequency and severity were deemed manageable, reinforcing the safety of HBOT for a broad patient demographic, including those with cardiac and pulmonary restrictions. Nonetheless, to ensure patient safety and comfort, the importance of vigilant monitoring and evaluation of side effects should not be understated.

### 4.6. Limitations and Future Direction

A significant limitation of the existing body of evidence is the absence of rigorous clinical trials and well-defined control groups, which impedes our ability to draw definitive conclusions regarding the benefits of HBOT and hinders our ability to make meaningful comparisons. None of the studies that were incorporated in this review exceeded Level III of the ASPS’ Evidence Rating Scale for Therapeutic Studies. This suggests that more high-quality randomized control trials are needed to confidently assess HBOT’s benefits in the treatment of NSM complications (Figure 3). We also acknowledge that HBOT is not the exclusive therapy for post-NSM treatment. Patients exploring alternatives to HBOT to enhance tissue viability and healing post-mastectomy might consider options such as topical nitroglycerin or diltiazem, local heat application, or reduction mammaplasty prior to mastectomy. These methods, which aim to enhance blood flow and tissue preconditioning, offer less invasive and infrastructure-dependent solutions. Discussing these alternatives with the patient can assist in determining the most suitable option for the individual circumstances [47,48,49,50]. There is a clear directive for future studies to determine the most effective timing and regimen for HBOT, evaluate its long-term impacts on patient recovery, and investigate its cost-effectiveness and accessibility. Such research is critical for the incorporation of HBOT into standardized care protocols following mastectomy. Moreover, while the findings suggest that HBOT may reduce the need for additional surgeries by decreasing compromised skin areas, the absence of control groups in several studies necessitates a cautious approach in interpreting these results.

## 5. Conclusions

In conclusion, HBOT has shown significant potential as an adjunct in post-surgical care for patients undergoing NSM, enhancing wound healing and reducing surgical complications. Its role in stimulating angiogenesis and improving tissue oxygenation underscores its therapeutic value. However, more rigorous clinical research is needed to refine HBOT protocols, including optimal timing, pressure settings, and patient eligibility. To fully integrate HBOT into standard care, future studies must address the long-term effects, cost-effectiveness, and accessibility. While preliminary findings are promising, a deeper exploration is essential to establish comprehensive guidelines for its effective and safe application in post-mastectomy recovery.

## Figures and Tables

**Table 1:** Presentation and characteristics of patients undergoing nipple-sparing mastectomy from case reports, observational case series, cohort studies, and retrospective studies.

Study ID	Study Design	Sample Size	BMI	Comorbidities (Number of Cases)	Number of Sessions	Days between Surgery and HBOT
Alperovich 2015 [24]	Case report	T = 1 Pre-HBOT (C) = 1 Post-HBOT (E) = 1	N/A	Healthy, nonsmoking, nondiabetic	30	2
Lotfi 2020 [25]	Observational case series	T = 7 Pre-HBOT (C) = 7 Post-HBOT (E) = 7	N/A	Not stated	Not stated	Not stated
Murphy 2017 [26]	Cohort study	T = 16 No HBOT (C) = 12 HBOT: Pre-Op (E) = 2 HBOT: Post-Op (E) = 2	N/A	Nonsmoking	Not stated	Not stated
Nasr 2023 [27]	Retrospective study	T = 17 Pre-HBOT (C) = 17 Post-HBOT (E) = 17	N/A	Smoker (1), nonsmoking (16)	19.4 ± 10.7	9.47 ± 12.7
Rajpal 2019 [28]	Retrospective study	T = 8 Pre-HBOT (C) = 8 Post-HBOT (E) = 8	25.6 ± 9.7	Hypertension (2), hyperlipidemia (3), smoker (1), seizures (1)	10	1
Shuck 2017 [29]	Cohort study	T = 13 Non-HBOT (C) = 5 HBOT (E) = 8	24.8	Smokers (2)	Not stated	Not stated
Copeland-Halperin 2016 [30]	Case report	T = 1 Pre-HBOT (C) = 1 Post-HBOT (E) = 1	N/A	Hypothyroidism	15	1

Abbreviations: ID, identification; BMI, body mass index; HBOT, hyperbaric oxygen therapy; T, treated; Pre-Op, pre-operation; Post-Op, post-operation; C, control; E, experimental; N/A, not applicable.

## Data Availability

No new data were created or analyzed in this study. Data sharing is not applicable to this article.
